# Retrolental cohesive ophthalmic viscoelastic injection for severe subluxated cataracts: a prospective study

**DOI:** 10.1186/s40942-024-00583-z

**Published:** 2024-09-19

**Authors:** Danilo Iannetta, Vito Romano, Nicola Valsecchi, Arianna Grendele, Simone Febbraro, Marco Marenco, Antonio Moramarco, Alessandro Lambiase, Luigi Fontana

**Affiliations:** 1 University of Rome La Sapienza Department of Organs of Sense, Rome, Italy; 2 Ophthalmology Unit, Dipartimento di Scienze Mediche e Chirurgiche, Alma Mater Studiorum University of Bologna, Bologna, Italy; 3 IRCCS Azienda Ospedaliero-Universitaria di Bologna, Bologna, Italy; 4 Department of Medical and Surgical Specialties, Radiological Sciences, and Public Health, Eye Clinic, University of Brescia, Brescia, 25121 Italy

**Keywords:** Complicated cataract surgery, Subluxated cataract, Cataract surgery, Surgical technique, Prospective study

## Abstract

**Background:**

To assess anatomical and functional outcomes of retrolental cohesive ophthalmic viscoelastic injection (“Viscolift technique”) in patients with severely subluxated cataracts.

**Methods:**

In the present prospective study, we included patients older than 18 years with severely subluxated cataracts and phacodonesis. Full medical history was obtained at the baseline ophthalmological assessment. A single 25-gauge valved trocar was inserted 4 mm from the limbus and a 27G angled cannula was introduced through the trocar into the retrolental space, while cohesive viscoelastic was progressively injected, in order to center and elevate the cataract to facilitate capsulorhexis. After complete phacoemulsification, a 3-piece intraocular lens (IOL) with a scleral fixated Cionni ring or FIL-SSF scleral fixated IOL was implanted. Patients follow-up interval was 6 months after surgery.

**Results:**

Thirteen eyes of 13 patients were enrolled in the study, mean age was 61.5 ± 9.4 years and 53.8% were females. The “Viscolift technique” resulted in centered and more stable cataracts in all cases (100%). After complete phacoemulsification, 61.5% of patients were implanted with a 3-piece IOL with Cionni ring, and 38.5% with a FIL-SSF scleral fixated IOL after complete 25G vitrectomy. Mean BCVA improved from 0.5 ± 0.1 LogMar (20/63 Snellen) to 0.1 ± 0.1 LogMar (20/25 Snellen) (*p* < 0.001) at the last follow-up. No major complications were noted.

**Conclusions:**

The “Viscolift technique” proved to be a safe and effective surgical approach for recentering and elevating subluxated cataracts, thus allowing the surgeon to perform an easier and better-centered capsulorhexis.

**Supplementary Information:**

The online version contains supplementary material available at 10.1186/s40942-024-00583-z.

## Background

The management of subluxated cataracts represents a surgical challenge, and a comprehensive preoperative evaluation is necessary to determine the most effective approach. For this purpose, it is essential to consider the degree of zonular weakness and whether the subluxation of the crystalline lens is congenital or acquired [1, 2]. Additionally, it is recommended to carry out a careful assessment of the anterior vitreous state before embarking on this type of surgery, as pathological conditions such as ocular trauma can alter the retrolental space and its associated structures [3]. 

Previous studies described different surgical techniques for the management via pars plana of sinking nuclei during cataract surgery in the presence of posterior capsular tears, such as the posterior-assisted levitation (PAL) technique. In brief, PAL consists of inserting a spatula downward via the pars plana with its tip inclined to the posterior pole of the eye, placing it behind the nucleus. The spatula lifts the partially dropped nucleus forward into the anterior chamber. Surgery is completed by extending the wound and expressing the nucleus or by phacoemulsification with the protection of a sheet glide [4]. In 2003, Chang and Packard described a modified PAL technique using Viscoat^®^ and the tip of its cannula to lift the nucleus or nuclear fragments into the anterior chamber [5]. Recently, Lifshitz et al. described the planned-PAL technique, where a spatula was inserted via the pars plana, the whole subluxated lens was lifted to the anterior chamber and then removed through a scleral tunnel [6]. 

Different devices can be employed to avoid complications due to the instability of the posterior capsular diaphragm. Some of the commercially available ones are the capsular hooks (MST Capsule Retractors, Chang Modification, MicroSurgical Technology Redmond, WA) for intraoperative purposes, the scleral fixated modified capsular tension ring (Cionni Capsular Tension Ring, Morcher GmbH) and the capsular tension segments (CTS, Morcher GmbH) or the second-generation capsular anchor for permanent purposes [7–9] During the management of subluxated cataracts, continuous curvilinear capsulorhexis (CCC) stands out as a crucial stage, as any discontinuity of the anterior capsule becomes a contraindication for the use of the abovementioned devices, undermining the in-bag implant. Recently, we described the “Viscolift technique,” a via pars plana approach to recenter and elevate the subluxated cataract in patients with an intact capsular bag and phacodonesis. This surgical technique consists of injecting cohesive viscoelastic in the retrolental space, achieving optimal conditions for capsulorhexis and complete phacoemulsification [10]. In our previous report, we illustrated the “Viscolift technique” in a single patient with a severely subluxated cataract, achieving complete phacoemulsification and preservation of the capsular bag in the absence of any intraoperative or postoperative complications. Hence, the main objective of the present study was to assess the anatomical and functional outcomes in patients with severely subluxated cataracts treated with the “Viscolift technique” with 6 months follow up.

## Methods

### Study population

Patients were recruited between October 2022 and September 2023 from an ongoing prospective study at the Unit of Ophthalmology, IRCSS University of Bologna. The study was conducted in accordance with the principles of the Declaration of Helsinki and was approved by the local Ethics Committee of the local health service of Bologna, Italy (Cod: 177/2023). Written informed consent was obtained from all the subjects included in the study. We included patients older than 18 years of age with severely subluxated cataracts (stage 3 at Hoffman classification [2]), phacodonesis, and a follow-up of at least 6 months. Patients with previous retinal detachment, corneal opacities, age-related macular degeneration, diabetic retinopathy, and a history of recurrent uveitis were excluded.

### Clinical assessment

A full medical history of medical therapies and comorbidities was obtained from each patient during the baseline assessment. The patients underwent a comprehensive ophthalmological evaluation both before and after cataract surgery.

All patients underwent eye examination, including assessment of best corrected visual acuity (BCVA) reported in logarithm of the minimum angle of resolution (LogMar), slit lamp examination of the anterior segment, Goldmann applanation tonometry, and indirect ophthalmoscopy. The cataract subluxation was graded according to the Hoffman classification [2]. 

Ocular biometry was performed using IOL MASTER 700 (Carl Zeiss Meditec AG, Jena, Germany), and each patient underwent an optical coherence tomography (OCT) examination of the macula before and after surgery using spectral domain (SD)-OCT (Heidelberg Spectralis, Heidelberg Engineering, Heidelberg, Germany). Last follow-up visit was at six months postoperatively.

### Surgical technique

**Fig. 1:**
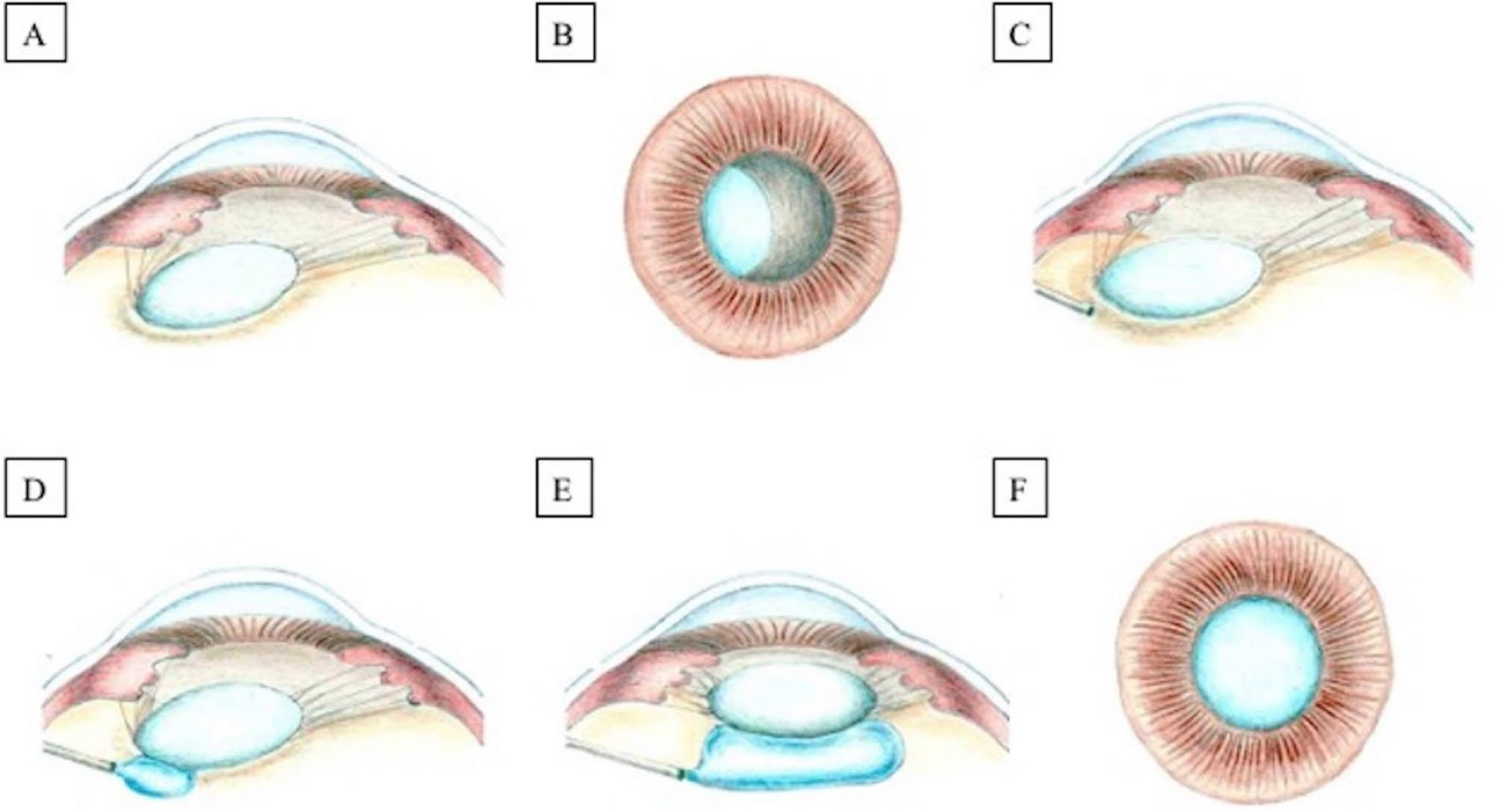
**(A)** and **(B)** Lens subluxation with a preserved capsular bag is depicted. **(C)** and **(D)** After insertion of a 27G angled cannula through a 25-gauge valved trocar 4 mm from the limbus, cohesive viscoelastic is injected in the retrolental space. **(E)** and **(F)** The lens is now more centered and stable, facilitating the capsulorhexis. (The image was previously published by our group [10].)

All the surgeries were performed by experienced surgeons. Surgery was performed under either subtenon or general anesthesia. Two corneal marks were placed at 0–180°, and a 300° conjunctival peritomy was performed to prepare for a scleral-fixated intraocular lens (IOL) implantation, whenever needed. After adequate diathermy, a single 25-gauge valved trocar was inserted 4 mm from the limbus where the cataract was primarily displaced. A nasal 1.2 mm paracentesis was performed to reduce intraocular pressure (IOP) in the anterior chamber. Subsequently, a 27G angled cannula was gradually introduced through the trocar into the retrolental space, while cohesive viscoelastic (Healon pro^®^-Johnson & Johnson, Inc.) was progressively injected to obtain a centered and more stable lens during the following surgical steps. The injection of viscoelastic began when the cannula was inserted in the trocar. The tip of the cannula was directed towards the lens anteriorly. This positioning enabled the cannula to pass through the hyaloid-capsular ligament and access the retrolental space of Berger. The dosage of viscoelastic injected ranged between 1 mL and 2 mL. However, there was no standard dosage, as it was determined by the intraocular pressure and the extent to which the lens could be recentered, while minimizing any trauma. See Fig. 1. Once the cataract was optimally positioned at the center, a complete capsulorhexis with a progressive implant of four capsular hooks (MST Capsule Retractors, Chang Modification, MicroSurgical Technology Redmond, WA) placed through four paracenteses to stabilize the bag was achieved. Complete phacoemulsification was performed with total removal of the epinucleus and cortex. According to the judgment of the surgeon, a Cionni ring with scleral sutures in Prolene 6 − 0 and a three-piece IOL or a sutureless scleral fixated intraocular lens (Soleko FIL SSF-IOL, Pontecorvo, Italy) after complete 25G vitrectomy were implanted. See **Supplemental Digital Content 1** that demonstrates the key steps of the surgical procedure.

### Statistical analysis

Normality was tested using the Shapiro–Wilk test. As the variables were normally distributed, they were reported as mean and standard deviation (SD), and parametric tests were used for statistical analysis. The Chi-square test was used to measure the association between two categorical variables. The paired T-test was used to evaluate the differences in BCVA, IOP, and refraction before and after surgery. *P* values < 0.05 were considered statistically significant. Statistical analysis was performed using IBM Statistical Package for Social Sciences version 26.

## Results

### Baseline assessment

A total of 13 eyes of 13 patients were included in the study; 6 patients were male (46.2%) and 7 were female (53.8%). The mean age of patients was 61.5 ± 9.4 years. Nine patients presented with a spontaneous subluxated cataract and phacodonesis (69.2%), two patients with a traumatic cataract (15.4%), one patient with subluxated cataract in a high myopic eye (7.7%), and one patient with a subluxated cataract associated to Marfan syndrome (7.7%) The mean baseline BCVA was 0.5 ± 0.1 LogMar (20/63 Snellen), and mean IOP was 17.3 ± 3.3 mmHg, whereas the mean refraction before surgery was − 1.1 ± 3.9 sphere diopters. See Table 1.

**Table 1:** Demographic data at the baseline assessment. BCVA = best corrected visual acuity; IOP = intraocular pressure SD = standard deviation

Demographic data	Patients (*n* = 13)
Age (years), mean ± SD	61.5 ± 9.4
Sex female, n (%)	7 (53.8%)
Causes of subluxated cataracts, n (%): - Spontaneous - Trauma - High myopia - Marfan syndrome	9 (69.2%) 2 (15.4%) 1 (7.7%) 1 (7.7%)
BCVA (LogMar), mean ± SD	0.5 ± 0.1
IOP (mmHg), mean ± SD	17.3 ± 3.3
Spherical equivalent (sphere diopters), mean ± SD	-1.1 ± 3.9

### Surgical approach

Surgery was performed under general anesthesia in five patients (38.5%), whereas in eight patients, subtenon anesthesia was performed (61.5%). The “Viscolift technique” allowed to obtain a centered and more stable cataract in all cases. In eight cases (61.5%), a 3-piece IOL with a Cionni ring was implanted. In five cases (38.5%), a complete 25G vitrectomy and a FIL-SSF scleral fixated IOL were preferred. No intraoperative or early postoperative complications were noted. The mean IOP was 18.2 3 ± 2.2 mmHg in the first post-operative day, and none of the patients required medications to decrease IOP in the following visits.

### Six-months follow-up

Six months after surgery, the mean BCVA improved to 0.1 ± 0.1 LogMar (20/25 Snellen) (*p* < 0.001). The mean refraction after surgery was − 0.5 ± 0.9 diopters, and the mean IOP was 16 ± 2 mmHg (*p* = 0.375 and *p* = 0.544, respectively). See Table 2. No major complications were noted. In two cases (15%), cystoid macular edema (CME) occurred after a mean of 14 ± 3.2 weeks after surgery. Of these two cases, one patient was previously implanted with a 3-piece IOL with a Cionni ring, whereas the other was implanted with a FIL SSF IOL fixated at the sclera. CME was managed with topical treatment in both cases, achieving complete resolution at the last follow-up visit.

**Table 2:** Ophthalmological assessment after 6 months from surgery. BCVA = best corrected visual acuity; IOP = intraocular pressure SD = standard deviation

Clinical data	Baseline	Follow-up at 6 months	*P* value
BCVA(LogMar), mean ± SD	0.5 ± 0.1	0.1 ± 0.1	**< 0.001**
IOP (mmHg), mean ± SD	17.3 ± 3.3	16 ± 2	*p* = 0.544
Spherical equivalent (sphere diopters), mean ± SD	-1.1 ± 3.9	-0.5 ± 0.9	*p* = 0.375

## Discussion

Surgical management of subluxated cataracts is challenging, and the choice of the best surgical approach depends on the severity of lens dislocation, the extent of zonular dialysis, and the underlying etiology. In 2013, Hoffman et al. proposed a new classification to assess the severity of the lens dislocation that considers the percentage of pupil in mydriasis left uncovered by the lens: 0 − 25% in minimal to mild subluxation, 25–50% in moderate subluxation and more than 50% in severe subluxation [2]. In cases from minimal to mild zonular dehiscence (less than 3 contiguous clock hours) and non-progressive conditions, a successful outcome can be obtained with a 3-piece or single-piece IOL in the capsular bag. In eyes with moderate to severe dehiscence ( more than 3 clock hours of zonular laxity), a permanent capsular support, such as a Cionni CTR or a Ahmed CTS, should be placed [2]. The determination of the most suitable capsular tension device should consider the intraoperative conditions and patient characteristics.

It is worth noting that the creation of a centered and well-sized capsulorhexis is essential for the effective placement of the abovementioned capsular tension devices since they are contraindicated if any discontinuity of the anterior capsule is present. Nonetheless, creating a well-centered capsulorhexis is challenging in subluxated cataracts due to zonular instability that reduces the counter-traction forces that facilitate the capsulotomy. Femto-laser capsulotomy has been proposed in recent years, as it minimizes the amount of stress on the already compromised zonules [11, 12]. However, it is severely limited when the anterior capsule is too tilted or too posteriorly displaced [11]. 

In cases of complete posterior dislocation of the crystalline lens in the vitreous cavity, pars plana vitrectomy is required, whereas soft nuclei can be removed by lensectomy and hard nuclei are better managed by phacofragmentation [13]. An alternative approach to reduce ultrasound energy and the risk of retinal injuries is the extraction of the hard nucleus through a corneoscleral limbal incision [14]. Other surgical techniques that can be employed are pars plana vitrectomy with intravitreal phacoemulsification [15], pars plana vitrectomy combined with perfluorocarbon liquid-assisted phacoemulsification [16, 17], and nitinol basket-assisted pars plana vitrectomy [18]. These surgical approaches have proven to be effective in removing the crystalline lens from the vitreous cavity. However, they carry an increased risk of intra-operative and post-operative complications such as iatrogenic retinal breaks, vitreous hemorrhage, vitreous incarceration and retinal detachment [19, 20]. 

Previous techniques have been described for the management via pars plana of sinking nuclei during cataract surgery in the presence of posterior capsular tears. Packard described the PAL technique, where a spatula is inserted downward via the pars plana with its tip inclined to the posterior pole of the eye, placed behind the nucleus and subsequently used to lift the partially dropped nucleus forward into the anterior chamber. Surgery is completed by extending the wound and expressing the nucleus or by phacoemulsification with the protection of a sheet glide [4]. Chang and Packard, in 2003, described a modified PAL technique using Viscoat^®^ where the tip of its cannula is used to lift the nucleus or nuclear fragments into the anterior chamber [5]. Lastly, Lifshitz et al. described the planned-PAL technique, where a spatula was inserted via the pars plana and used to lift the whole subluxated lens to the anterior chamber and then remove it through a scleral tunnel [6]. 

In the present study, we described the anatomical and functional outcomes of the “Viscolift technique.” This technique consists of injecting a cohesive viscoelastic substance into the retrolental space to raise and center the lens in cases of severely subluxated cataracts and this facilitates the surgeon in performing a centered capsulorhexis, preserving the capsular bag. In addition, it offers posterior support to stabilize the anterior chamber intraoperatively and, since the use of Healon is gentler than the spatula, it allows preservation of the posterior capsule [10]. The main finding of the present study was that the “Viscolift technique” effectively resulted in elevation and centration of the lens in all cases. Furthermore, we observed a significant improvement in BCVA and refraction in all cases 6 months after surgery, with an improvement from 0.5 ± 0.1 LogMar (20/63 Snellen) pre-operatively to 0.1 ± 0.1 LogMar (20/25 Snellen) six months after surgery, outlining this technique’s efficacy in managing severely subluxated cataracts. Chee at al. reported an improvement in BCVA from a median of 0.6 logMar pre-operatively to 0.2 logMar at one year in patients with severely subluxated cataracts treated with Femto-laser assisted capsulotomy [11]. Also, Chee and Jap reported a visual acuity of 20/40 or better after one year in almost 95% of eyes with traumatic subluxated cataracts treated with capsular tension devices [1]. Therefore, our results suggest that the “Viscolift technique” is at least as effective as previous surgical methods in terms of improvement in visual acuity in patients with severely subluxated cataracts.

According to the results of our study, the “Viscolift technique” showed to be a viable option in the presence of zonular disinsertion. It is worth noting that an essential prerequisite for the success of this surgical approach is a preoperative condition of phacodonesis, as the mobility itself facilitates the elevation of the lens with the viscoelastic substance. Therefore, it is crucial to assess the degree of phacodonesis before surgery to determine the appropriateness of this technique. Cohesive viscoelastic agents are particularly indicated for this surgical technique due to their ability to create and sustain an optimal space, as well as to facilitate lens recentering in instances of severe subluxation. Their rheological properties, marked by long-chain molecules, high molecular weight, and elevated viscosity enhance cohesion and ensure effective space maintenance, thereby facilitating precise lens repositioning [21]. Another result of the present study was that we did not encounter any complications intraoperatively or during the 6-month follow-up period, such as increased IOP, retinal tears, or retinal detachments, with the exception of two cases of CME that were successfully treated with topical medications. Furthermore, in none of the patients a pars plana lensectomy or vitrectomy for dropped nucleus or fragments or the use of fragmatome was necessary. Following surgery, a temporary rise in intraocular pressure may occur as a result of viscoelastic substances used during the procedure. This increase in pressure typically peaks 4 to 7 h after surgery and usually normalizes within a few days. In case of IOP increase, it can be managed effectively with either topical or systemic medications [21]. 

This study has several limitations such as the small number of patients included and the single-center study assessment. Therefore, a multicenter study with a larger number of cases should be performed to confirm the effectiveness of the technique. Additionally, further studies are needed to compare the “Viscolift technique” with other surgical approaches for severe subluxated cataracts, in order to understand its potential advantages over alternative surgical methods.

In conclusion, the management of subluxated cataracts represents a challenge, and the choice of the most suitable approach or device to address every specific situation is a demanding task for the surgeon. The results of the present study showed that the “Viscolift technique” is a safe and effective surgical approach for re-centering and elevating subluxated cataracts to facilitate capsulorhexis. Therefore, surgeons should consider this approach as a viable option for the management of patients with severe crystalline subluxation, intact capsular bags, and phacodonesis.

## Electronic supplementary material

Below is the link to the electronic supplementary material.

Supplementary Material 1

## Data Availability

No datasets were generated or analysed during the current study.

## Abbreviations

PAL: Posterior-assisted levitation
CTS: Capsular tension segments
CCC: Continuous curvilinear capsulorhexis
BCVA: Best corrected visual acuity
LogMar: Logarithm of the minimum angle of resolution
OCT: Optical coherence tomography
IOL: Intraocular lens
IOP: Introcular pressure
SD: Standard deviation
CME: Cystoid macular edema
